# Supplementary material for: A comparison between predetermined and self-selected approaches in resistance training: effects on power performance and psychological outcomes among elite youth athletes
Source: PeerJ. 2020 Nov 12;8:e10361. doi: 10.7717/peerj.10361 (PMC7666817; doi:10.7717/peerj.10361)
Supplement: Data S1 [file peerj-08-10361-s002.docx]

***Supplemental file***

The inter-day reliability of the power outputs, ROF and PACES responses to both experimental conditions was examined using Pearson correlation analysis. Values ≤0.1, between 0.1-0.3, between 0.3-0.5, between 0.5-0.7, between 0.7-0.9 and >0.9 were interpreted as trivial, small, moderate, large, very large, and nearly perfect.^1^ To complement the correlation analysis, the level of agreement between the sessions was examined with Bland-Altman bias estimates. The 95% CI of the mean difference was used to determine systematic bias.^2^ Reliability scores of all variables are reported in the table below. The majority of the performance scores were reliable except for the bench press exercise P_mean_ scores during the predetermined condition. Conversely, low reliability but no systematic biases were observed for the ROF responses and PACES scores across all exercises in both conditions.

**Table S1. Reliability scores of all the collected measures**

LoA: limits of agreement; P_mean_: mean propulsive power; ROF: rate of fatigue; PACES: physical activity enjoyment scale

|  | Reliability Measures | | | |
| --- | --- | --- | --- | --- |
|  | **Pearson correlation**  **r** | | **95% of measures within 2 SD of LoA mean** | |
|  | **Predetermined** | **Self-selected** | **Predetermined** | **Self-selected** |
| Power outputs  Squat P_mean_ | 0.9 | 0.6 | Yes | Yes |
| Jump squat P_mean_ | 1.0 | 0.5 | Yes | Yes |
| Bench press P_mean_ | 0.9 | 0.7 | No | Yes |
| Bench throw P_mean_ | 0.9 | 0.8 | Yes | Yes |
| ROF  Baseline  Back squat  Jump squat  Bench press  Bench throw | 0.3  0.2  0.6  0.3  0.6 | 0.3  0.3  0.2  0.1  0.1 | Yes  Yes  Yes  Yes  Yes | Yes  Yes  Yes  Yes  Yes |
| PACES | 0.5 | 0.1 | Yes | Yes |
|  | | | | |

**References**

1. Hopkins WG. Linear models and effect magnitudes for research, clinical and practical applications. *Sportscience*. 2010;14:49-57.
2. Leech, N, Onwuegbuzie, A. A call for greater use of nonparametric statistics. 2003.
